# Attachment and Borderline Personality Features: The Mediating Roles of Hypomentalizing and Epistemic Mistrust

**DOI:** 10.1002/cpp.70185

**Published:** 2025-11-27

**Authors:** Yağızcan Kurt, Tobias Nolte, Patrick Luyten, Janet Feigenbaum, Brooks King‐Casas, Judy Leibowitz, Steve Pilling, P. Read Montague, London Personality and Mood Disorder Research Network, Peter Fonagy

**Affiliations:** ^1^ Department of Clinical, Educational and Health Psychology University College London London UK; ^2^ Anna Freud London UK; ^3^ Department of Psychology Istanbul Medeniyet University Istanbul Turkey; ^4^ Faculty of Psychology and Educational Sciences University of Leuven Leuven Belgium; ^5^ Fralin Biomedical Research Institute, Department of Psychology Virginia Tech Roanoke Virginia USA; ^6^ Camden and Islington Psychological Therapies Services Camden & Islington NHS Foundation Trust London UK

**Keywords:** attachment, borderline personality features, epistemic mistrust, mentalizing

## Abstract

Insecure attachment is a well‐established risk factor for the emergence of borderline personality features (BPF), encompassing identity disturbance, affective instability, problematic interpersonal relationships and self‐harming behaviours. From a mentalizing‐based perspective, BPF may develop through the interplay of hypomentalizing, defined as a reduced capacity to understand and reflect on one's own and others' mental states, and epistemic mistrust (EM), the diminished capacity to trust communicated information. Both processes are shaped by attachment anxiety and avoidance. However, research investigating the mechanisms linking attachment to BPF remains limited. This study examined the parallel mediating roles of hypomentalizing and EM in the associations between attachment dimensions and BPF. Data were drawn from a large combined clinical and community sample of 1129 participants (291 men, 819 women, 17 transgender individuals and 2 categorized as ‘other’; mean age = 32.88 years, SD = 10.90). Participants completed the Experiences in Close Relationships—Revised (ECR‐R); the Reflective Functioning Questionnaire–54 (RFQ‐54); the Epistemic Trust, Mistrust and Credulity Questionnaire (ETMCQ); and the Borderline Features Scale of the Personality Assessment Inventory (PAI‐BOR). Parallel multiple mediation analyses indicated that both hypomentalizing and EM significantly mediated the relationships between (a) attachment anxiety and BPF and (b) attachment avoidance and BPF. Supplementary analyses showed that these pathways did not differ across diagnostic groups, but EM was expressed most strongly in those participants who met criteria for borderline personality disorder (BPD), where it differentiated the group from clinical (depression and anxiety disorders) and community comparisons. These findings support the theoretical basis for mentalization‐based interventions, highlighting the importance of improving mentalizing and reducing mistrust in communication to foster adaptive social learning. Nonetheless, the cross‐sectional design and reliance on self‐report measures limit causal inference and introduce potential bias. Future research should employ longitudinal designs to further evaluate the proposed model.

## Summary

- Attachment anxiety and avoidance contribute to borderline personality features (BPF) through impaired mentalizing and reduced trust in communicated information.
- Assessing hypomentalizing and epistemic mistrust in individuals with insecure attachment may help identify mechanisms underlying identity disturbance, affective instability, relational difficulties and impulsivity.
- Conceptualizing BPF in terms of disrupted social learning and epistemic mistrust can enhance case formulation and guide treatment planning.
- Interventions may be strengthened by explicitly targeting both mentalizing deficits and epistemic mistrust to support relational functioning in individuals with BPF.

## 1 Introduction

Borderline personality disorder (BPD) is characterized by instability in identity, interpersonal relationships and affect regulation, alongside impulsivity (APA 2013). The clinical severity is considerable: About 5.9% of affected individuals die by suicide (Temes et al. 2019), and many continue to experience marked impairment despite treatment (Cristea et al. 2017; Woodbridge et al. 2022). These challenges highlight the importance of clarifying the developmental and cognitive–affective processes that contribute to borderline pathology.

Originally described in psychoanalytic theory as a level of personality between neurosis and psychosis (Kernberg and Caligor 2005), borderline pathology was later formalized as a categorical diagnosis in DSM‐III. In recent years, however, diagnostic systems have shifted again to dimensional perspectives. The ICD‐11 emphasizes levels of personality functioning, and DSM‐5 Section III introduced the Alternative Model for Personality Disorders (AMPD), which combines a focus on impairments in levels of personality functioning and maladaptive trait domains to conceptualize personality pathology. The strongest evidence for the AMPD comes from research on BPD, where AMPD‐defined borderline pathology has demonstrated equal or superior antecedent, concurrent and predictive validity compared with the categorical diagnosis, alongside higher reliability and greater clinical utility in identifying impairments and treatment targets (Sharp et al. 2025). Supporting this shift, BPD criteria load strongly on a general factor of personality pathology (Sharp et al. 2015), validated instruments such as the LPFS and STiP‐5.1 provide reliable assessment (Morey 2017; Hutsebaut et al. 2017) and large‐scale studies show that impairments in personality functioning predict a broad range of psychopathology and long‐term outcomes (Kerber et al. 2024). Together, these findings support a dimensional view of borderline personality features (BPF).

At the same time, dimensional assessment does not preclude categorical distinctions. For example, ICD‐11 classifies personality disorders by severity within a dimensional framework but also includes a *borderline pattern specifier*. Similarly, instruments such as the Personality Assessment Inventory—Borderline Personality Subscale (PAI‐BOR) capture clinically distinct profiles: high BPF scores map onto hallmark domains of BPD and differentiate it from other conditions (Stein et al. 2007; Venta et al. 2018). Elevated PAI‐BOR scores are linked to emotion regulation difficulties (Cheavens et al. 2012), trauma exposure (Riemann et al. 2024) and poorer long‐term outcomes in community samples (Trull et al. 1997; Lazarus et al. 2016; Howard et al. 2022). They are also associated with cognitive–affective biases and disability outcomes (Kelly Grealy et al. 2022; Reynolds and Tragesser 2019). Moreover, heterogeneity within BPD is patterned, with subgroups differing systematically in trauma, comorbidity and symptom patterns (Antoine et al. 2023).

What is less clear are the mechanisms that give rise to these distinct profiles. Mentalizing‐based approaches highlight the capacity to interpret behaviour in terms of underlying mental states as an important factor in this respect (Fonagy and Luyten 2016). This ability develops in early attachment relationships (Luyten et al. 2020) and disruptions in attachment experiences may compromise mentalizing, leaving individuals prone to either hypermentalizing—being overly confident about the mental state of others—or hypomentalizing, marked by low levels of often biased mentalizing reflecting difficulties forming coherent representations of the self and others (Fonagy et al. 2016; McLaren et al. 2022).

Recent extensions of this model also highlight the importance of epistemic trust in this regard, the capacity to treat communicated knowledge as trustworthy, relevant and personally applicable (Fonagy et al. 2015). Reduced epistemic trust, or epistemic mistrust (EM), may undermine social learning and adaptation (Nolte et al. 2023). Yet, although EM has been proposed as central to borderline difficulties in combination with hypomentalizing, to the best of our knowledge, neither of these factors has been studied simultaneously as a mechanism linking attachment and BPF.

This study addresses this research gap through a parallel mediation model exploring the indirect effects of hypomentalizing and EM on the association between attachment and BPF in a large (*N* = 1129), combined sample comprising individuals diagnosed with personality disorders (BPD or antisocial personality disorder [ASPD]), depressive or anxiety disorders and a community sample. While, as noted, hypermentalizing may also be relevant in this context (McLaren et al. 2022), we focused exclusively on hypomentalizing, as the psychometric validity of the hypermentalizing subscale in the instrument used—the Reflective Functioning Questionnaire (RFQ) (Fonagy et al. 2016)—has been called into question (Müller et al. 2022).

### 1.1 Theoretical Framework

Insecure attachment represents a well‐documented risk factor for BPF. Disrupted caregiving—manifested as neglect, abuse or inconsistent responsiveness—constitutes core elements of insecure attachment, potentially fostering the development of central BPF (Kurt and Çakır 2025). Such attachment disruptions frequently involve *a lack of marked mirroring*, wherein caregivers inadequately reflect the child's internal states, thereby failing to communicate emotional understanding and regulatory support (Fonagy et al. 2002). Consequently, the child may struggle to form a coherent self‐concept, lacking stable opportunities to develop internalized representations of their own mental states through attuned caregiver interactions.

Adult attachment is commonly conceptualized along two continuous dimensions: attachment anxiety and attachment avoidance (Mikulincer and Shaver 2007). Attachment anxiety reflects heightened concern about relational security and emotional responsiveness, often accompanied by preoccupation with the availability of close others, fear of rejection and a tendency towards reassurance seeking and emotional dependence. In contrast, attachment avoidance involves discomfort with intimacy and a strong preference for independence, marked by efforts to maintain emotional distance and a tendency to downplay the importance of close relationships.

Attachment theory posits that early relational experiences shape cognitive–affective representations, or internal working models, of self and others, guiding interpretations and responses to interpersonal interactions (Bowlby 1988). Inconsistent caregiving can prompt hyperactivation of the attachment system, typified by anxious attachment, where individuals escalate efforts to secure consistent emotional responsiveness (Mikulincer and Shaver 2007). Conversely, persistent emotional rejection or unavailability may lead to attachment system deactivation, characteristic of avoidant attachment, where individuals suppress emotional needs to mitigate distress (Mikulincer and Shaver 2007). Both attachment dimensions may contribute to disruptions in identity formation, emotional instability and turbulent relationships—core features of BPD—as supported by a meta‐analysis linking adult attachment to BPD (Smith and South 2020). Although insecure attachment is highly prevalent among individuals with BPD (around 90%; Levy et al. 2005), the vast majority of those with insecure attachment do not meet criteria for any personality disorder.

One potential mechanism linking attachment and BPF is hypomentalizing. Anxious attachment is associated with hyperactivating strategies, characterized by intensified efforts to seek care and reassurance, typically accompanied by elevated emotional arousal (Luyten et al. 2020). This reactive, affectively charged form of mentalizing—comparable with a fight‐or‐flight response—lacks the reflective depth necessary for comprehending the complexities of self and others (Kurt and Çakır 2025). In contrast, avoidant attachment involves negative internal working models, characterized by low expectations of others' availability or support (Luyten et al. 2020). In this context, hypomentalizing may function defensively, shielding individuals from distressing interpersonal cues or potential rejection. A compromised reflective capacity undermines the formation of a stable self‐concept, impeding the coherent understanding of personal thoughts, emotions and intentions, thereby fostering identity fragmentation (Luyten et al. 2020). Difficulties interpreting others' intentions exacerbate interpersonal instability and emotional dysregulation. Severe impairments in reflective functioning can precipitate prementalizing modes, such as the *teleological mode*, wherein observable behaviour alone is deemed meaningful (Luyten et al. 2020). Consequently, individuals may engage in impulsive or self‐harming behaviours, bypassing internal mental states in favour of immediate, concrete responses to distress.

Another proposed mechanism linking disrupted attachment to BPF is EM. Fonagy et al. (2015) propose that secure attachment establishes conditions under which communication is perceived as trustworthy, thus facilitating social learning. Core relational processes—including caregiver sensitivity, contingent responsiveness and marked mirroring—communicate that others are credible sources of information about oneself and the environment. Conversely, insecure attachment contexts, such as those involving emotional neglect or abuse, foster EM (Campbell et al. 2021; Kampling et al. 2022; Liotti et al. 2023). Within these environments, children adopt a suspicious stance towards communication, interpreting interpersonal input as potentially harmful (Nolte et al. 2023). As an adaptive response, they inhibit trust in received messages, shaping enduring internal working models of others into adulthood (Kurt 2025). Among individuals with attachment anxiety, this manifests as compulsive reassurance seeking coupled with persistent doubts about its authenticity (Fonagy et al. 2015), whereby their need for validation is overshadowed by fears of rejection. Those with attachment avoidance, however, habitually disregard interpersonal communication as inherently unreliable. This generalized mistrust prevents internalization of social feedback, critical for coherent and adaptive identity formation (Kurt 2025). EM further disrupts interpersonal relationships by hindering accurate social cue interpretation, adaptive responsiveness and effective conflict resolution, contributing to chronic misunderstanding and affective instability. Moreover, emotion regulation is compromised as opportunities for interpersonal coregulation become inaccessible. Without trust in others' input, individuals may resort to impulsive behaviours or self‐harm as immediate, non‐symbolic strategies to alleviate distress (Fonagy 2000).

### 1.2 Previous Evidence and the Current Study

A recent meta‐analysis confirmed that both attachment anxiety and avoidance are associated with BPF, with attachment anxiety showing a stronger association (*r* = 0.48) than avoidance (*r* = 0.30), underscoring the clinical relevance of attachment dimensions in BPF (Smith and South 2020). In addition, Badoud et al. (2018) identified mentalizing as a key mechanism linking insecure attachment to BPD, highlighting the role of social‐cognitive vulnerabilities in the disorder. Previous studies have also shown that EM is associated with elevated levels of general psychopathology (Campbell et al. 2021; Liotti et al. 2023). Moreover, emerging evidence suggests that epistemic trust may function as a psychological bridge between early adversity and various forms of psychopathology, including PTSD and complex PTSD (Kampling et al. 2022), general psychopathology (Campbell et al. 2021), psychological maladjustment (Tironi et al. 2024) and BPF (Knapen et al. 2025). However, to date, no study has specifically examined EM as a mediator between adult attachment and BPF, while also controlling for hypomentalizing.

A recent study by Knapen et al. (2025) explored a related pathway, testing attachment, mentalizing and epistemic trust as parallel mediators between childhood trauma and BPD symptoms. Our approach differs by conceptualizing EM and mentalizing as proximal consequences of current adult attachment dimensions, rather than as direct outcomes of childhood trauma (Fonagy et al. 2015; Luyten et al. 2020). This allows for a focused investigation of how adult relational schemas influence epistemic trust and mentalizing capacity, which may in turn contribute to BPF. This framework aligns with recent findings showing that the association between childhood trauma and mentalizing difficulties becomes non‐significant when adult attachment is accounted for (Kurt and Çakır 2025), emphasizing the central role of adult attachment in these psychological processes.

**FIGURE 1:**
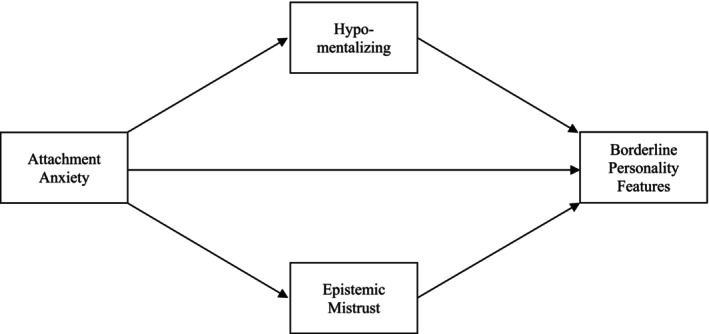
Mediating roles of hypomentalizing and epistemic mistrust in the relationship between attachment anxiety and borderline personality features.

**FIGURE 2:**
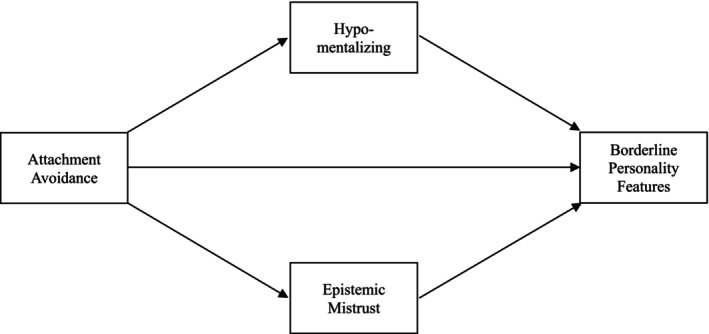
Mediating roles of hypomentalizing and epistemic mistrust in the relationship between attachment avoidance and borderline personality features.

In this study, we examined two parallel mediation models using a cross‐sectional dataset from a large, combined sample comprising individuals diagnosed with personality disorders (BPD or ASPD), depressive or anxiety disorders and a community sample. We investigated the mediating roles of hypomentalizing and EM in the associations between (a) attachment anxiety and BPF and (b) attachment avoidance and BPF. Secure attachment is theorized to support the development of relational and regulatory capacities by facilitating the accurate interpretation and use of interpersonal feedback (Fonagy et al. 2015; Luyten et al. 2020). In contrast, insecure attachment—marked by hyperactivating or deactivating strategies—may undermine these processes, increasing vulnerability to impaired social understanding and mistrust in others' communication. Heightened EM and reduced mentalizing may, in turn, impair the internalization of interpersonal feedback, hinder the repair of relational ruptures and limit the use of others for emotional regulation. Accordingly, we hypothesized that both hypomentalizing and EM would mediate the associations between adult attachment dimensions and BPF. The parallel mediation models are illustrated in Figures 1 and 2.

In response to reviewer comments, we also examined whether these pathways were moderated by sample type, comparing individuals with BPD, those with depression and anxiety disorders and community participants. This was done in order to address whether the mechanisms linking attachment, hypomentalizing and EM are specific to BPD or reflect broader transdiagnostic processes. We further tested whether EM differed categorically across these groups, given its proposed centrality to BPD (Fonagy et al. 2015), and explored which variables, including attachment dimensions, hypomentalizing and EM, best differentiated between the three groups.

## 2 Method

### 2.1 Transparency and Openness

This study was pre‐registered on the Open Science Framework on 24 November 2024 (https://osf.io/wtf8k/?view_only=5eb4aa843ee34eef9a2c53abd1eb31bb). In response to reviewer comments, we also conducted additional post hoc analyses. These included moderated mediation with sample type as a moderator, a one‐way analysis of variance testing differences in EM across the three groups, and a discriminant function analysis. These analyses were carried out to explore whether the models operate transdiagnostically and to identify which variables most clearly distinguish between groups.

### 2.2 Participants

An a priori Monte Carlo power analysis for indirect effects (Schoemann et al. 2017) determined the required sample size for detecting parallel mediation effects with 80% statistical power. Sample sizes from 200 to 1000, in increments of 10, were tested using 5000 replications and 20,000 Monte Carlo draws per replication (95% CI, random seed = 1717). Predicted effect sizes, informed by prior literature, are detailed in Data S1. The analysis indicated a necessary sample of 700 participants for detecting mediation by hypomentalizing and EM between attachment anxiety and BPF and 590 for mediation involving attachment avoidance, both achieving 80% power (95% CI [0.79, 0.82]).

Participants were recruited from three sources in Greater London: (a) specialist personality disorder and probation services (BPD or ASPD), (b) NHS Talking Therapies services (for depression or anxiety) and (c) community settings (e.g., universities and online platforms). Recruitment began in 2013 and remains ongoing; this dataset has supported multiple research projects.

Inclusion criteria were age between 16 and 65 years and fluency in English. Exclusion criteria comprised schizophrenia or recent psychosis, learning disabilities and neurological disorders. Additional inclusion criteria for the BPD/ASPD group were a confirmed or suspected diagnosis and current placement on a therapy waiting list or psychoeducational programme. For participants from NHS Talking Therapies, criteria included significant depressive symptoms and eligibility for low‐ or high‐intensity psychological interventions.

Diagnostic procedures differed across recruitment sources. For the BPD/ASPD group, diagnostic status was confirmed using the Structured Clinical Interview for DSM‐IV Axis II Disorders (SCID‐II; First et al. 2016). For the HC group, participants were screened using the Standardised Assessment of Personality—Abbreviated Scale (SAPAS; Moran et al. 2003). In the earlier years of recruitment, those scoring ≥ 4 were additionally administered the SCID‐II, but since ~2019, SCIDs were no longer conducted. Thus, HC eligibility in later years relied solely on SAPAS screening. For the depression/anxiety group, eligibility was established within NHS Talking Therapies services. Participants were assessed by NHS clinicians as suitable for 1:1 low‐ or high‐intensity therapy and were excluded if bipolar or psychotic disorders were indicated. A small number of early participants in this group also received SCIDs, but for most participants diagnostic status was based on NHS assessment records and self‐reported presenting problems.

The final sample comprised 1129 participants: 291 men, 819 women, 17 transgender individuals and 2 categorized as ‘other’. Mean age was 32.88 years (SD = 10.90). Detailed demographic and clinical characteristics are presented in Table 1.

**TABLE 1:** Sociodemographic characteristics of the total sample.

Sociodemographic characteristic	Frequency	
	*n*	%
Gender		
Men	291	25.8
Women	819	72.5
Transexual/transgender	17	1.5
Other	2	0.2
Education level		
No qualifications	38	3.4
Other qualifications	38	3.4
Vocational Level 1, GCSE (< 5 A*–C) or equivalent	62	5.5
GCSE (5+ A*–C), Vocational Level 2 or equivalent	84	7.4
A‐level, Vocational Level 3 or equivalent	255	22.6
Higher education or professional/vocational equivalent	410	36.3
Postgraduate education or professional/vocational equivalent	241	21.3
Subsample		
Borderline personality disorder	143	12.7
Community sample	485	43.0
Depressive or anxiety disorder	501	44.4

### 2.3 Measures

#### 2.3.1 Adult Attachment

Adult attachment was measured with the 36‐item Experiences in Close Relationships Scale (ECR‐R; Fraley et al. 2000). Items were rated on a 7‐point Likert scale (1 = *strongly disagree*, 7 = *strongly agree*). The scale assesses attachment anxiety and avoidance (18 items each) in romantic relationships, with higher scores indicating greater attachment insecurity. The ECR‐R has robust psychometric properties (Sibley et al. 2005). Cronbach's alpha in the current study was 0.94 for both attachment anxiety and avoidance.

#### 2.3.2 Hypomentalizing

Mentalizing was assessed using the 54‐item RFQ (Fonagy et al. 2016), rated on a 7‐point Likert scale (1 = *strongly disagree*, 7 = *strongly agree*). The RFQ comprises two subscales: Uncertainty (reflecting an extreme lack of understanding of mental states) and Certainty (excessive confidence regarding mental state understanding). This study utilized the Uncertainty subscale, with higher scores indicating greater hypomentalizing. The RFQ shows good test–retest reliability and internal consistency (Fonagy et al. 2016). Cronbach's alpha for the Uncertainty subscale in this study was 0.90. We also conducted a sensitivity analysis using the eight‐item version of the RFQ, which yielded similar overall findings. As the 54‐item version is the original instrument, results are reported based on this version.

#### 2.3.3 Epistemic Mistrust

EM was measured with the 15‐item ETMCQ, rated on a 7‐point Likert scale (1 = *strongly disagree*, 7 = *strongly agree*). Among the ETMCQ's three subscales—Trust, Mistrust and Credulity—the current study specifically used the Mistrust subscale, with higher scores indicating greater EM. The ETMCQ has demonstrated strong validity and reliability (Campbell et al. 2021). Cronbach's alpha for the Mistrust subscale in this study was 0.72.

#### 2.3.4 BPF

BPF were measured using the 24‐item PAI‐BOR, with items rated on a 4‐point Likert scale (0 = *false*, 3 = *very true*). The scale comprises four subscales—Affective Instability, Negative Relationships, Identity Problems and Self‐Harm—which collectively assess diverse facets of borderline pathology. This study utilized the total summed score across all items, with higher scores indicating greater severity. The PAI‐BOR has demonstrated robust psychometric properties across varied populations (Morey 1991; Stein et al. 2007). Cronbach's alpha for the total scale in this study was 0.92.

### 2.4 Procedure

A cross‐sectional design was employed. Participants from the BPD, ASPD, and HC groups attended at least two in‐person assessment sessions at University College London, completing behavioural tasks, self‐report questionnaires, and structured personality interviews. Participants recruited from NHS Talking Therapies services (with depression or anxiety) participated remotely, completing self‐report questionnaires and one behavioural task, but not personality interviews. All participants provided informed consent and received compensation of £10 per hour, with additional incentives based on task performance.

The dataset originated from the broader project titled ‘Probing Social Exchanges—A Computational Neuroscience Approach to Borderline and Anti‐Social Personality Disorder’, and its substudy ‘Major Depressive Disorder—A Computational Psychiatry Approach’. Ethical approvals were granted by the Wales REC (12/WA/0283) for the primary BPD/ASPD/HC study and the London Queen Square REC (16/LO/0077) for the substudy.

### 2.5 Data Analysis

Data were analysed using SPSS Version 28 and the PROCESS v4.2 macro (Hayes 2017). Pearson's correlations were calculated to examine associations between variables, while *t* tests and one‐way ANOVA assessed group differences across demographic categories.

Parallel multiple mediation analyses (PROCESS Model 4) tested the hypothesized mediating pathways, using 5000 bootstrap samples and 95% confidence intervals. Indirect effects were interpreted as statistically significant when bootstrap confidence intervals did not include zero.

In response to reviewer comments, non–pre‐registered moderated mediation models (PROCESS Model 59) tested whether indirect pathways differed across diagnostic groups (BPD, depression/anxiety and community). A one‐way ANOVA examined group differences in EM, followed by post hoc Bonferroni comparisons. Finally, a discriminant function analysis evaluated which variables best distinguished between the three groups.

## 3 Results

### 3.1 Descriptive Statistics and Bivariate Correlations

Table 2 presents descriptive statistics and intercorrelations among study variables. Age significantly correlated with attachment anxiety (*r* = −0.15, *p* < 0.001), EM (*r* = −0.07, *p* < 0.001) and BPF (*r* = −0.19, *p* < 0.001). Years of education were negatively correlated with attachment anxiety (*r* = −0.08, *p* = 0.011), attachment avoidance (*r* = −0.13, *p* < 0.001), hypomentalizing (*r* = −0.15, *p* < 0.001), EM (*r* = −0.15, *p* < 0.001) and BPF (*r* = −0.17, *p* < 0.001).

**TABLE 2:** Descriptive statistics and correlations for study variables.

Variable	*n*	*M*	SD	1	2	3	4	5	6	7
1. ECR‐R‐AN	1129	3.70	1.39	—						
2. ECR‐R‐AV	1129	3.20	1.27	0.43***	—					
3. RFQ_U	1129	15.79	12.98	0.33***	0.26***	—				
4. EM	1129	4.43	1.06	0.41***	0.34***	0.38***	—			
5. PAI‐BOR	1129	32.13	15.21	0.59***	0.35***	0.48***	0.55***	—		
6. Age	1129	32.88	10.90	−0.15***	0.05	−0.02	−0.07*	−0.19***	—	
7. Edu	1129	15.14	4.06	−0.08*	−0.13***	−0.15***	−0.15***	−0.17***	0.00	—

Abbreviations: ECR‐R‐AN, Experiences in Close Relationships—Revised—Attachment Anxiety; ECR‐R‐AV, Experiences in Close Relationships—Revised—Attachment Avoidance; Edu, mean years in education; PAI‐BOR, Personality Assessment Inventory—Borderline Scale; RFQ_U, Reflective Functioning Questionnaire—Uncertainty Scale.

* *p* < 0.05.

** *p* < 0.01.

*** *p* < 0.001.

We additionally examined potential gender differences on primary variables. Earlier in recruitment, response options were male, female, transsexual and transgender. In later years, the options were simplified to male, female and other (with free‐text specification). For reporting purposes, we combined noncisgender responses into a single ‘other’ category (*N* = 19). Within this category, 17 participants identified as transgender (with no further specification available regarding transition direction), and 2 participants provided other free‐text responses. Due to limited sample sizes for transgender (*n* = 17) and ‘other’ (*n* = 2) categories, analyses compared men (*n* = 291) and women (*n* = 819) only, using independent samples *t* tests. Men reported significantly higher hypomentalizing scores (*M* = 17.23, SD = 13.49) compared with women (*M* = 15.18, SD = 12.76), *t*(1108) = 2.32, *p* = 0.021, 95% CI [0.32, 3.78], *d* = 0.16.

### 3.2 Mediating Roles of Hypomentalizing and EM Between Attachment Anxiety and BPF

A parallel multiple mediation analysis (Hayes 2017) examined whether hypomentalizing and EM mediated the relationship between attachment anxiety and BPF. Gender, age and mean years of education were included as covariates. All reported coefficients and effects are unstandardized.

**FIGURE 3:**
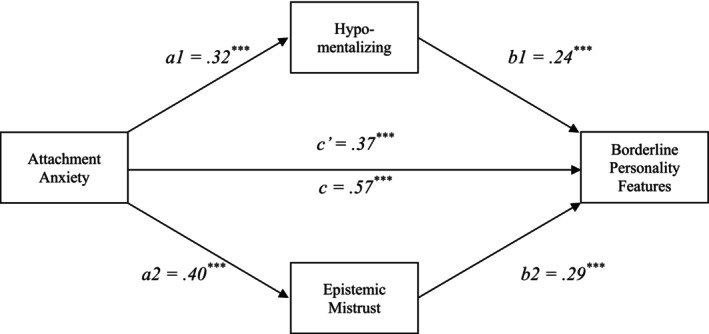
Parallel multiple mediating variable analysis: the roles of hypomentalizing and epistemic mistrust between attachment anxiety and borderline personality features. *Note:* The coefficients are standardized effects. *a*_1_ = Attachment Anxiety → Hypomentalizing; *a*_2_ = Attachment Anxiety → Epistemic Mistrust; *b*_1_ = Hypomentalizing → Borderline Personality Features; *b*_2_ = Epistemic Mistrust → Borderline Personality Features; *c* = total effect; *c*′ = direct effect.

As shown in Figure 3, there was a significant indirect effect of attachment anxiety on BPF through hypomentalizing, controlling for EM (indirect effect = 0.86, bootSE = 0.11, 95% CI [0.64, 1.08]). This indicates that greater attachment anxiety was associated with higher hypomentalizing, which in turn was linked to increased BPF, independently of EM.

A significant indirect effect was also observed through EM, controlling for hypomentalizing (indirect effect = 1.27, bootSE = 0.14, 95% CI [1.01, 1.55]). Thus, higher attachment anxiety was associated with greater EM, which was related to higher levels of BPF, independently of hypomentalizing.

The overall mediation model was significant, *F*(6, 1122) = 211.63, *p* < 0.001, accounting for 53.1% of the variance in BPF (*R* ^2^ = 0.53). Contrast analysis of indirect effects demonstrated a significantly stronger indirect pathway through EM compared with hypomentalizing (contrast = 0.41, bootSE = 0.18, 95% CI [0.06, 0.77]). Detailed model results are provided in Table 3.

**TABLE 3:** Unstandardized indirect and direct effects in the parallel mediation model of hypomentalizing and epistemic mistrust between attachment anxiety and borderline personality features.

Path	Effect	bootSE	95% CI
ANX → HYPO → BPF	0.8595	0.1121	[0.6437, 1.0928]
ANX → EM → BPF	1.2695	0.1371	[1.0051, 1.5471]
Total indirect effect	2.1290	0.1723	[1.7934, 2.4754]
Direct effect	4.1143	0.2544	[3.6152, 4.6134]
Total effect	6.2433	0.2618	[5.7296, 6.7570]
Contrast (EM–HYPO)	0.4100	0.1818	[0.0603, 0.7737]

*Note:* Results were considered significant when the 95% confidence intervals (CIs) did not include zero.

Abbreviations: ANX, Attachment Anxiety total scores; bootSE, bootstrapped standard error; BPF, Borderline Personality Features scores; CI, confidence interval; EM, Epistemic Mistrust scores; HYPO, Hypomentalizing scores.

As a follow‐up, we tested a moderated parallel mediation model (PROCESS Model 59) to examine whether the indirect pathways from attachment anxiety to BPF via hypomentalizing and EM differed across the BPD, depression/anxiety and community samples. Gender, age and education were again included as covariates.

Attachment anxiety significantly predicted both hypomentalizing (*b* = 2.16, *p* = 0.008) and EM (*b* = 0.15, *p* = 0.018). Hypomentalizing was a consistent predictor of BPF (*b* = 0.16, *p* = 0.003), with significant indirect effects across BPD (indirect effect = 0.35, 95% CI [0.03, 0.75]), depression/anxiety (indirect effect = 0.35, 95% CI [0.17, 0.58]) and community (indirect effect = 0.58, 95% CI [0.34, 0.86]) samples.

The indirect effect through EM was non‐significant in the BPD group (indirect effect = 0.19, 95% CI [−0.07, 0.64]) but significant in both the depression/anxiety group (indirect effect = 0.57, 95% CI [0.32, 0.88]) and the community group (indirect effect = 0.68, 95% CI [0.42, 0.98]). Importantly, indices of moderated mediation were non‐significant for both mediators, indicating that although indirect effects differed descriptively between groups, their magnitudes did not differ reliably. Overall, the mediational pathways from attachment anxiety to BPF through hypomentalizing and EM thus appear broadly transdiagnostic.

### 3.3 Mediating Roles of Hypomentalizing and EM Between Attachment Avoidance and BPF

A parallel multiple mediation analysis (Hayes 2017) similarly examined the roles of hypomentalizing and EM as mediators linking attachment avoidance to BPF. Gender, age and mean years of education were included as covariates. All coefficients and effects reported are unstandardized.

**FIGURE 4:**
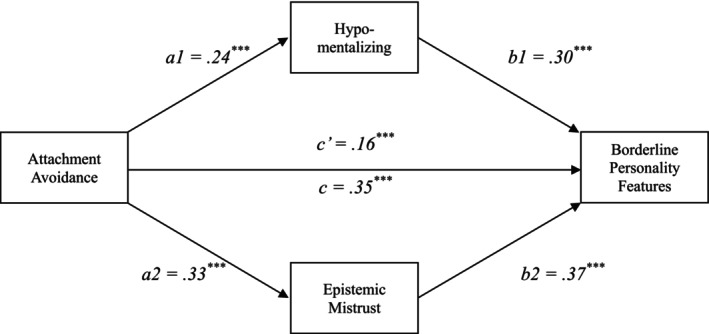
Parallel multiple mediating variable analysis: the roles of hypomentalizing and epistemic mistrust between attachment avoidance and borderline personality features. *Note:* The coefficients are standardized effects. *a*_1_ = Attachment Avoidance → Hypomentalizing; *a*_2_ = Attachment Avoidance → Epistemic Mistrust; *b*_1_ = Hypomentalizing → Borderline Personality Features; *b*_2_ = Epistemic Mistrust → Borderline Personality Features; *c* = total effect; *c*′ = direct effect.

As illustrated in Figure 4, attachment avoidance had a significant indirect effect on BPF via hypomentalizing, controlling for EM (indirect effect = 0.87, bootSE = 0.13, 95% CI [0.63, 1.15]). Thus, greater attachment avoidance was associated with increased hypomentalizing, which, in turn, predicted higher BPF, independently of EM.

Additionally, a significant indirect effect emerged through EM, controlling for hypomentalizing (indirect effect = 1.45, bootSE = 0.16, 95% CI [1.14, 1.76]). Therefore, higher attachment avoidance was linked to greater EM, subsequently related to elevated BPF, independently of hypomentalizing.

The overall model was significant, *F*(6, 1122) = 147.21, *p* < 0.001, explaining 44.1% of the variance in BPF (*R* ^2^ = 0.44). Indirect effect contrast analysis revealed that the EM‐mediated pathway was significantly stronger than the hypomentalizing‐mediated pathway (contrast = 0.58, bootSE = 0.20, 95% CI [0.18, 0.96]). Full model details are presented in Table 4.

**TABLE 4:** Unstandardized indirect and direct effects in the parallel mediation model of hypomentalizing and epistemic mistrust between attachment avoidance and borderline personality features.

Path	Effect	bootSE	95% CI
AVO → HYPO → BPF	0.8707	0.1304	[0.6325, 1.1468]
AVO → EM → BPF	1.4546	0.1592	[1.1406, 1.7636]
Total indirect effect	2.3254	0.2128	[1.9070, 2.7422]
Direct effect	1.7861	0.2896	[1.2178, 2.3543]
Total effect	4.1114	0.3270	[3.4699, 4.7530]
Contrast (EM–HYPO)	0.5839	0.1985	[0.1838, 0.9573]

*Note:* Results were considered significant when the 95% confidence intervals (CIs) did not include zero.

Abbreviations: AV, Attachment Avoidance total scores; bootSE, bootstrapped standard error; BPI, Borderline Personality Inventory total scores; CI, confidence interval; CTQ, Childhood Trauma Questionnaire total scores; MEN, Mentalizing Questionnaire total scores.

As a follow‐up, we tested a moderated parallel mediation model (PROCESS Model 59) to examine whether the indirect pathways from attachment avoidance to BPF via hypomentalizing and EM differed across the BPD, depression/anxiety and community samples. Gender, age and education were again included as covariates.

Attachment avoidance significantly predicted EM (*b* = 0.17, *p* = 0.006) but not hypomentalizing (*b* = 0.85, *p* = 0.29). Both hypomentalizing (*b* = 0.19, *p* = 0.001) and EM (*b* = 2.16, *p* = 0.036) significantly predicted BPF. The indirect effect through hypomentalizing was significant in the depression/anxiety (indirect effect = 0.42, 95% CI [0.19, 0.71]) and community samples (indirect effect = 0.56, 95% CI [0.29, 0.86]) but not in the BPD group (indirect effect = 0.16, 95% CI [−0.21, 0.64]).

The indirect effect through EM was significant across all groups: BPD (indirect effect = 0.38, 95% CI [0.00, 0.91]), depression/anxiety (indirect effect = 0.64, 95% CI [0.34, 0.99]) and community (indirect effect = 0.79, 95% CI [0.50, 1.12]).

Indices of moderated mediation were non‐significant for both mediators, suggesting that the strength of these indirect pathways did not reliably differ between groups. Thus, the mediational pathways from attachment avoidance to BPF via hypomentalizing and EM also appear transdiagnostic.

### 3.4 Analysis of Variance and Discriminant Function Analysis

As the mediation models were found to be transdiagnostic, we next examined which variables distinguished the diagnostic groups. Given that EM has been proposed as particularly elevated in BPD (Fonagy et al. 2015) and was central to the present models, we first conducted a one‐way ANOVA comparing BPD, depression/anxiety and community samples. Results indicated a significant group effect, *F*(2, 1126) = 106.90, *p* < 0.001, *η* ^2^ = 0.16, reflecting large between‐group differences. Post hoc Bonferroni tests showed that the BPD group (*M* = 5.26, SD = 0.83) reported significantly higher EM than both the depression/anxiety group (*M* = 4.01, SD = 0.96, *p* < 0.001) and the community group (*M* = 4.61, SD = 1.02, *p* < 0.001). The community group also reported significantly higher EM than the depression/anxiety group (*p* < 0.001).

To extend this analysis, a discriminant function analysis was conducted using attachment anxiety, attachment avoidance, hypomentalizing and EM as predictors of group membership. Two discriminant functions were extracted. Function 1 accounted for 95.2% of the between‐group variance (eigenvalue = 0.30, canonical *R* = 0.48, Wilks' Λ = 0.76, *χ* ^2^(8) = 313.94, *p* < 0.001), and Function 2 accounted for 4.8% (eigenvalue = 0.02, canonical *R* = 0.12, Wilks' Λ = 0.99, *χ* ^2^(3) = 17.01, *p* < 0.001).

The structure matrix indicated that Function 1 was defined primarily by EM (0.79), attachment anxiety (0.72) and hypomentalizing (0.64), with attachment avoidance contributing more weakly (0.48). Function 2 was defined mainly by hypomentalizing (0.61) and negatively by EM (−0.49). Group centroids suggested that Function 1 differentiated the BPD group (1.20) from the depression/anxiety (−0.50) and community (0.15) groups, whereas Function 2 provided only minor distinctions.

Taken together, the results showed that EM, attachment anxiety and hypomentalizing most clearly distinguished the BPD group from the other groups, consistent with theoretical accounts that emphasize EM and mentalizing difficulties as central to borderline pathology.

## 4 Discussion

In this study, we investigated the mediating roles of hypomentalizing and EM in the relationships between (a) attachment anxiety and BPF and (b) attachment avoidance and BPF. As hypothesized, both hypomentalizing and EM showed significant indirect effects in both mediation models, with EM emerging as the stronger mediator in each case.

These findings align with prior research identifying mentalizing impairments as mediators linking attachment insecurity to borderline personality pathology (Badoud et al. 2018). They are also consistent with recent evidence showing that EM is associated with elevated levels of general psychopathology and that both hypomentalizing and EM mediate the associations between childhood maltreatment and various psychopathological outcomes (Campbell et al. 2021; Kampling et al. 2022; Liotti et al. 2023; Tironi et al. 2024), including BPD symptoms (Knapen et al. 2025). However, our finding that EM had a stronger mediating effect than hypomentalizing contrasts with results reported by Knapen et al. (2025), who found that EM accounted for only 17% of the mediation effect linking childhood trauma to BPD symptoms, compared with 22% for attachment anxiety and 42% for hypomentalizing. Notably, Knapen et al. used the Questionnaire of Epistemic Trust (QET) to assess EM, which is different from the measure used in the present study.

Supplementary analyses provided additional insight into both transdiagnostic and potential disorder‐specific patterns. The moderated mediation analyses indicated that the indirect pathways through hypomentalizing and EM were invariant across groups, suggesting that these are transdiagnostic mechanisms, consistent with theoretical assumptions of the mentalizing approach (Fonagy et al. 2015; Luyten et al. 2020). Yet, at the same time, a one‐way ANOVA showed that EM was significantly higher in BPD compared with depression/anxiety and community groups, with a quarter of BPD participants scoring above 6 on a 7‐point scale. In addition, the discriminant function analysis demonstrated that EM, attachment anxiety and hypomentalizing distinguished BPD from other groups, supporting the assumption that these features are particularly central in the dynamics of individuals with BPD features. Together, these results suggest that while hypomentalizing and EM are relevant across diagnoses, they are disproportionately elevated and more defining in BPD, consistent with theoretical accounts positioning mistrust of social communication as central to borderline pathology (Fonagy et al. 2015; Nolte et al. 2023).

Individuals with attachment anxiety typically employ hyperactivating strategies—marked by intense, persistent efforts to solicit care and reassurance from others, frequently driven by elevated emotional arousal (Luyten et al. 2020). Chronic hyperarousal disrupts controlled mentalizing, causing a shift towards automatic mentalizing, where individuals struggle to form nuanced representations of their own and others' mental states. Instead, they rely on simplified, reactive interpretations of interpersonal cues—strategies potentially adaptive in emotionally charged, fight‐or‐flight situations (Kurt and Çakır 2025). However, impaired mentalizing compromises identity stability, preventing coherent integration of self‐concepts and contributing to confusion around personal values, emotions and objectives. Difficulties interpreting social cues also impair interpersonal functioning and emotion regulation, as misunderstandings of others' intentions exacerbate conflict and inhibit effective emotional coregulation. Thus, these mentalizing impairments likely heighten vulnerability to BPF.

Attachment anxiety may similarly promote EM. Research indicates individuals with insecure attachment strategies frequently reject information that contradicts their established internal working models (Mikulincer and Arad 1999). Among anxiously attached individuals, these internal models often encompass negative self‐perceptions (viewing oneself as unworthy) and pessimistic expectations about others (anticipating rejection) (Mikulincer and Shaver 2007). Consequently, even genuine reassurance may be doubted, with supportive communications perceived as insincere (Campbell et al. 2021). This pervasive mistrust reinforces negative self‐beliefs and destabilizes relationships, as reassurance‐seeking behaviour co‐occurs paradoxically with rejection of received validation. Over time, such dynamics may foster a self‐fulfilling prophecy resistant to corrective experiences: relentless reassurance seeking may alienate others, inadvertently confirming rejection fears. Even when others express genuine care, EM may lead individuals to disregard supportive gestures and withdraw interpersonally, creating relationship strain. Deprived of trustworthy external feedback necessary for adaptive self‐concept revision (Fonagy et al. 2015; Nolte et al. 2023), individuals become entrenched in rigidly negative self‐perceptions and relationship instability, thereby increasing susceptibility to BPF.

In contrast, attachment avoidance typically involves deactivating strategies, wherein individuals suppress attachment needs to minimize closeness (Luyten et al. 2020). In this context, hypomentalizing may serve defensively, diminishing awareness of mental states to avoid emotional vulnerability (Kurt and Çakır 2025). Consequently, these individuals may struggle with self‐awareness, remaining disconnected from their emotions, with interpersonal interactions frequently characterized by conflict—contributing significantly to BPF. Similarly, EM associated with attachment avoidance often manifests as a dismissive interpersonal stance, pre‐emptively rejecting others' input due to perceptions of unreliability or untrustworthiness. This aligns with avoidantly attached individuals' negative internal working models, portraying others as unavailable, unreliable and rejecting (Mikulincer and Shaver 2007). Such individuals might test relationships by pushing others away, observing if genuine care persists. However, due to persistent EM mediated through rigid internal working models, authentic support is frequently dismissed as insincere. Over time, this may lead to increasing emotional detachment and relational isolation, as individuals fail to use social feedback adaptively to develop coherent self‐concepts. Rigid self‐reliance reinforces a self‐fulfilling prophecy: emotional distance and scepticism push others away, confirming their beliefs that others are inherently untrustworthy. The inability to form secure, trusting relationships limits adaptive integration of interpersonal feedback, exacerbating vulnerability to BPF.

The discrepancy between our finding—that EM was the stronger mediator—and Knapen et al.'s (2025) finding that hypomentalizing predominated may reflect methodological differences, including measurement tools, model specification and sample characteristics. Knapen et al. reported a strong correlation (*r* = 0.73) between hypomentalizing, measured via the eight‐item RFQ, and BPD symptoms, measured with the MSI‐BPD (Zanarini et al. 2003). Some RFQ items conceptually overlap with MSI‐BPD symptoms, which may have inflated associations. Similarly, different instruments were used to assess EM: We employed the Mistrust subscale of the ETMCQ, whereas Knapen et al. used the QET. While conceptually related, these measures differ in scope, as noted by Knapen et al. (2024). Lastly, our larger sample (*N* = 1129 vs. *N* = 245) likely improved estimate stability and statistical power.

Taken together, findings that indirect pathways did not differ across groups, combined with evidence that EM was the strongest mediator, compared with hypomentalizing, in linking attachment dimensions to BPF, and that individuals with BPD showed the highest levels of EM compared with both other groups, support a dual conceptualization of EM. Consistent with recent mentalizing approaches, hypomentalizing and EM seem to operate as transdiagnostic vulnerability factors, making individuals more vulnerable to a wide range of psychological problems (Fonagy et al. 2015; Luyten et al. 2020). On the other hand, EM also emerges as a feature more characteristic of BPD, differentiating it from both clinical and non‐clinical groups. This interpretation aligns with Sharp et al. (2015), who demonstrated that BPD criteria load strongly onto a general factor of personality pathology, positioning BPD as a prototype for understanding personality dysfunction, while also being consistent with accounts emphasizing EM as central to borderline pathology (Fonagy et al. 2015). EM may therefore represent both a general mechanism of persistent psychopathology and a characteristic marker of BPD.

### 4.1 Clinical Implications

Our finding that the first model accounted for 53% of the variance in BPF, and the second model accounted for 44%, suggests clinicians should carefully assess attachment dimensions, hypomentalizing and EM when evaluating risk for BPF and designing psychosocial interventions. Specifically, our results suggest that interventions targeting the improvement of mentalizing abilities, and the reduction of EM may be particularly beneficial for individuals exhibiting insecure attachment patterns and BPF.

The supplementary analyses further reinforce these implications. Although hypomentalizing and EM operated as transdiagnostic mechanisms, EM was most elevated in BPD and most strongly distinguished this group from both clinical and community comparisons. This suggests that while interventions addressing EM may have broad clinical value, they are especially critical in treating BPD, where high levels of mistrust and hypomentalizing can undermine the therapeutic alliance. The observation that a quarter of individuals with BPD scored above 6 on a 7‐point scale for EM highlights the possibility of ceiling effects, suggesting that mistrust may be so entrenched in this group that it constrains responsiveness to new or alternative social input unless addressed directly in therapy.

One promising clinical approach to reducing EM while enhancing mentalizing involves the process of recognition, whereby the therapist explicitly demonstrates accurate understanding of the patient's perspective and concerns (Fisher et al. 2023). When patients feel genuinely understood, they may become increasingly curious about the mind of the individual who understands them (Nolte et al. 2023). Such curiosity can facilitate enhanced mentalizing, enabling individuals to more accurately interpret social cues and reduce tendencies towards hostile or maladaptive attributions.

Improved mentalizing capacities can further support a shift from a defensive stance of interpersonal mistrust towards a more adaptive and accurate view of social environments, enabling patients to perceive others' intentions as benign rather than threatening and to discriminate appropriately when that is not the case. This attributional shift can strengthen interpersonal trust, allowing individuals to more effectively utilize social support and environmental resources to enhance overall well‐being. Consistent with this perspective, a recent meta‐analysis identified interpersonal mistrust as a core difficulty among individuals with BPD (Kurt et al. 2025), highlighting mistrust as a significant barrier to social learning and adaptive functioning within this population.

Moreover, EM may significantly contribute to the recognized challenges of treating persistent psychopathology (Fonagy et al. 2015). Without establishing foundational trust, therapeutic alliances often remain fragile, potentially increasing early dropout rates among patients who are already difficult to engage (Berghuis et al. 2021). Regardless of therapeutic modality, fostering trust is essential to encourage openness to therapeutic input and prevent negative treatment outcomes. Therefore, interventions prioritizing trust establishment and facilitating patients' experiences of being genuinely understood may enhance mentalizing, reduce EM and ultimately improve treatment outcomes for individuals presenting with BPF.

### 4.2 Limitations and Future Directions

Several limitations should be acknowledged. Firstly, the cross‐sectional design restricts our ability to make causal inferences or establish definitive temporal relationships among variables. Although our mediation models were guided by theoretical assumptions, cross‐sectional analyses have inherent limitations concerning the temporal ordering of variables (Maxwell et al. 2011). Future research should employ longitudinal designs to clarify the directionality and causal relationships between attachment dimensions, hypomentalizing and EM.

Secondly, measurement error remains a potential issue, as different instruments may yield varying results even within similar populations. Structural equation modelling (SEM) using latent constructs could mitigate measurement error by enabling more precise estimation of underlying constructs. For instance, EM could be modelled as a latent variable incorporating indicators from both the ETMCQ and the QET, enhancing measurement robustness and reliability. In addition, the distribution of EM scores in the BPD group suggested possible ceiling effects, with a quarter of participants scoring above 6 on a 7‐point scale. This restricted variability may have attenuated associations in that group, underscoring the need for measures that are sensitive across the full range of mistrust.

Thirdly, alternative mediators not examined in this study may better capture the mechanisms underlying the observed relationships. Our mediation models were constructed based on theoretical considerations, but future research should consider alternative explanatory mechanisms. For instance, we did not include hypermentalizing in our models due to measurement limitations. Future research could incorporate a robust measure of hypermentalizing to examine whether its inclusion alters the findings. Additionally, replicating our findings across diverse samples using different instruments would enhance generalisability. The predominance of female participants in our sample further restricts the applicability of results to other populations, highlighting the need for broader representation in future research.

Fourthly, we did not include epistemic credulity in our models, as we believe the theoretical rationale linking epistemic credulity to the study variables differs from that of EM and warrants separate investigation. For instance, the pathway from epistemic credulity to BPF may be more closely related to identity diffusion, as credulous individuals tend to accept incoming information indiscriminately, which may contribute to unstable or shifting values and a lack of coherent identity. Future studies may consider including epistemic credulity to further explore these distinct mechanisms.

Another limitation is that the current sample was primarily drawn from treatment‐seeking individuals in personality disorder or mental health services in Greater London, as well as a community sample, which may limit the generalizability of the findings to broader populations or individuals from more diverse cultural and socioeconomic backgrounds. Although our sample included community participants, a substantial proportion were treatment‐seeking individuals whose experiences and characteristics may differ markedly from those of the general population who do not seek treatment. Notably, both EM and attachment styles are likely to be culturally embedded constructs, and the measures used in this study have been primarily validated in Western contexts. Future research should assess whether these constructs and their corresponding instruments demonstrate equivalent validity and relevance in culturally diverse populations.

Another limitation involves reliance on self‐report measures, which are subject to biases such as social desirability, mood effects and limited self‐awareness (Podsakoff et al. 2003). Assessing mentalizing via self‐report specifically may inadequately capture the complexity and subtlety of mentalizing processes (Fonagy et al. 2016). The exclusive use of self‐report methods also increases the risk of common method variance, potentially inflating observed associations between variables. Future studies should integrate alternative measurement methods, such as behavioural assessments or laboratory tasks, to reduce bias and provide a more comprehensive and nuanced understanding of the constructs. A further limitation is that the ECR‐R does not capture disorganized or fearful attachment, which has been linked to personality difficulties (Fonagy et al. 2015). More generally, self‐report questionnaires have limitations in assessing attachment, particularly in individuals with personality disorder features; future research should employ measures or designs that allow disorganized attachment to be assessed.

Finally, the discriminant function analysis was exploratory, and the variables identified as distinguishing between groups should be replicated in independent samples before firm conclusions are drawn, particularly as these analyses were not pre‐registered and were conducted in response to reviewer comments.

## 5 Conclusion

In conclusion, these results highlight the potential clinical value of psychosocial interventions aimed at enhancing mentalizing and reducing EM, particularly among individuals exhibiting high attachment insecurity. Such interventions may be instrumental in improving relational functioning and reducing vulnerability to BPF. The findings further suggest that while EM operates as a transdiagnostic mechanism, it seems particularly central in individuals with BPD features, underscoring its importance as both a general and specific treatment target.

## Funding

Yağızcan Kurt was sponsored by the Republic of Türkiye Ministry of National Education for his postgraduate studies. Yağızcan Kurt is grateful to the Republic of Türkiye Ministry of National Education for funding his PhD. This work was supported by a National Institute for Health Research (NIHR) Senior Investigator Award (NF‐SI‐0514‐10157) awarded to Peter Fonagy. The authors also acknowledge funding by NIH‐NIDS Grant 5R01NS092701‐03 and a Wellcome Trust Principal Research Fellowship awarded to P. Read Montague.

## Conflicts of Interest

The authors declare no conflicts of interest.

## Supporting information


**Data S1:** Supporting Information.

## Data Availability

The data that support the findings of this study are available from the corresponding author upon reasonable request.
